# Wheat growth monitoring and yield estimation based on remote sensing data assimilation into the SAFY crop growth model

**DOI:** 10.1038/s41598-022-09535-9

**Published:** 2022-03-31

**Authors:** Chunyan Ma, Mingxing Liu, Fan Ding, Changchun Li, Yingqi Cui, Weinan Chen, Yilin Wang

**Affiliations:** 1 School of Surveying and Land Information Engineering, Henan Polytechnic University, Jiaozuo, 454000 Henan China; 2 Zhangzhou Institute of Surverying and Mapping, Zhangzhou, 363000 Fujian China

**Keywords:** Plant sciences, Environmental sciences

## Abstract

Crop growth monitoring and yield estimate information can be obtained via appropriate metrics such as the leaf area index (LAI) and biomass. Such information is crucial for guiding agricultural production, ensuring food security, and maintaining sustainable agricultural development. Traditional methods of field measurement and monitoring typically have low efficiency and can only give limited untimely information. Alternatively, methods based on remote sensing technologies are fast, objective, and nondestructive. Indeed, remote sensing data assimilation and crop growth modeling represent an important trend in crop growth monitoring and yield estimation. In this study, we assimilate the leaf area index retrieved from Sentinel-2 remote sensing data for crop growth model of the simple algorithm for yield estimation (SAFY) in wheat. The SP-UCI optimization algorithm is used for fine-tuning for several SAFY parameters, namely the emergence date (D_0_), the effective light energy utilization rate (ELUE), and the senescence temperature threshold (STT) which is indicative of biological aging. These three sensitive parameters are set in order to attain the global minimum of an error function between the SAFY model predicted values and the LAI inversion values. This assimilation of remote sensing data into the crop growth model facilitates the LAI, biomass, and yield estimation. The estimation results were validated using data collected from 48 experimental plots during 2014 and 2015. For the 2014 data, the results showed coefficients of determination (R^2^) of the LAI, biomass and yield of 0.73, 0.83 and 0.49, respectively, with corresponding root-mean-squared error (RMSE) values of 0.72, 1.13 t/ha and 1.14 t/ha, respectively. For the 2015 data, the estimated R^2^ values of the LAI, biomass, and yield were 0.700, 0.85, and 0.61, respectively, with respective RMSE values of 0.83, 1.22 t/ha, and 1.39 t/ha, respectively. The estimated values were found to be in good agreement with the measured ones. This shows high applicability of the proposed data assimilation scheme in crop monitoring and yield estimation. As well, this scheme provides a reference for the assimilation of remote sensing data into crop growth models for regional crop monitoring and yield estimation.

## Introduction

As one of the essential food crops, wheat is widely planted worldwide, with a total planting area exceeding 200 million hectares, and more than one third of the world population using wheat as the main food ingredient^1,2^. Wheat is also one of the main food crops in China, where the wheat total planting area and yield come just next to those of rice and corn. Several biophysical variables can be used to assess the growth of wheat and other crops including mainly the leaf area index (LAI) and the biomass. Indeed, these two variables represent the material basis for crop yield formation. As well, the dynamic changes in those two parameters during different growth periods are closely related to the full crop yield formation. Therefore, LAI and biomass monitoring can provide essential information for analyzing energy balance and flow in terrestrial ecosystems. In particular, wheat yield estimation enables timely understanding of the changing trends of wheat harvesting and production, provides a reference for macro-level control and formulation of trade policies, and helps ensure food security through the formulation and implementation of policies for food pricing, circulation, and storage.

The wheat LAI, biomass, and yield metrics have been traditionally obtained through field measurement methods. Although these methods generally have a high accuracy, they may not be appropriate for monitoring wheat growth in widely distributed countries with complex topography like China. Meanwhile, wheat growth is not only related to intrinsic physiological and structural characteristics, but is also influenced by environmental factors (such as climate, hydrology, and soil patterns) as well as social, economic, and technological factors. Therefore, wheat growth patterns exhibit wide temporal and spatial variations, and thus manual monitoring methods for such patterns are labor-intensive and generally inefficient. In addition, human observers of crop growth should have high expertise and crop knowledge. Hence, outcomes of manual monitoring methods are highly subjective and not suitable for large-scale monitoring. Alternatively, methods based on remote sensing are fast, objective, and non-destructive, and hence offer unique advantages in monitoring physiological and biochemical crop parameters and estimating crop yield. In recent years, remote sensing technology was widely used in estimating the crop LAI, biomass, and yield.

For LAI and biomass estimation, Huang et al*.*, Su et al*.*, and Fieuzal et al.^3–5^ used the canopy red light band (680–760 nm), LiDAR, SAR C HH and L HH band data. Then, they calculated the red edge position, amplitude, and area as well as other parameters. In addition, they extracted vertical structure parameters, analyzed their correlation with LAI, and finally constructed LAI estimation models for rice, corn, wheat and other crops. Furthermore, Liu et al*.*, Li et al*.*, and Wang et al*.*^6–8^ exploited remote sensing data collected from environmental satellites (such as MODIS, ASTER, SPOT5). Then, vegetation index maps were combined with LAI measurements to create PROSAIL statistical models for dynamic LAI estimation in maize and wheat crops. Thenkabail et al*.*^9^ showed that crop biomass is negatively correlated with spectral reflectance in the red-light band (620–700 nm), and positively correlated with spectral reflectance in the near-infrared band (740–1100 nm). So, a biomass inversion model was established based on these observations. Takahashi et al*.*^10^ investigated the relationship between canopy spectral reflectance and rice biomass in the spectral range of 400–1100 nm, and hence established a biomass inversion model based on partial least squares regression. Blackard et al*.*^11^ used MODIS and Landsat TM image data with regression methods to devise biomass inversion algorithms and draw a national biomass distribution map in the United States. Gao et al*.*^12^ used environmental satellite image data and ground measurements to construct biomass inversion models based on multiple linear regression, and employed the high-precision models for biomass estimation. Li et al*.*, Zheng et al*.*, Zhang et al*.*^13–15^ fused spectral reflectance and image data of wheat canopy for wheat biomass estimation. In addition, raw spectral data was employed in several methods to filter sensitive bands, build spectral or vegetation index maps, analyze index correlation with crop biomass, and finally build biomass inversion models. For example, Casanova et al*.*^16^ analyzed rice spectral reflectance data and calculated several relevant vegetation indices, namely the ratio vegetation index (RVI), the normalized difference vegetation index (NDVI), the vertical vegetation index (VVI), and the weight difference vegetation index (WDVI). An analysis of the correlation between these vegetation indexes and rice biomass showed that the VVI and WDVI are relatively strong predictors for rice biomass estimation. Moreover, Chen et al*.*, Barati et al*.*, Irykna et al*.*, and Newnham et al*.*^17–20^ used remote sensing data to build regression models for estimating crop biomass from vegetation indices, evaluated the estimation results, and generated maps of the spatial biomass distribution in the studied areas. Bao et al*.*^21^ explored the feasibility of multiple-scale estimation of the biomass of winter wheat using the normalized difference vegetation index (NDVI), the enhanced vegetation index (EVI), and the normalized vegetation index (NVI). As well, Tan et al*.*, Gao et al*.*, and Lu et al*.*^22–24^ utilized UAV digital images, RADARSAT-2 data, HJ-1 A/B data, and Landsat TM data to construct spectral and vegetation index maps for estimating and mapping the biomass of soybean, wheat, and other crops. Liu et al*.*^25^ combined spectral parameters, texture factors, and terrain factors with ground measurements in order to construct an inversion model for wheat biomass estimation.

For crop yield estimation, numerous remote sensing approaches have been reported as well. For instance, Wu et al*.*^26^ collected spectral data of soybean canopy and associated yield measurements during multiple growth periods. This data was used to construct a comprehensive soybean yield estimation model across multiple growth periods (R^2^ = 0.68). Gao et al*.*, Ren et al*.*, and Akhand et al.^27–29^ used MODIS data, AVHRR images, HJ satellite images, SPOT4 and TM5 images to construct maps of the normalized difference vegetation index (NDVI) and the vegetation health index (VHI). These vegetation indices were used as predictors within regression models for corn and potato yield estimation. Li et al*.* and Sun et al.^30,31^ used MODIS data and Landsat image data to calculate the NDVI and LAI of wheat and grapes, and estimate the yield of these crops using multiple linear regression models. Chen et al*.* and Ou et al.^32,33^ analyzed HJ satellite time-series data and extracted characteristic parameters of the NDVI change rates across different growth periods for winter wheat, rice, corn, and soybean. These parameters were used to create yield estimation models and find periods with the best estimated yield for the four crop types. Song et al*.* and Zhao et al.^34,35^ used hyperspectral data measured by a GER1500 spectrometer and an unmanned aerial vehicle (UAV) to construct maps of the ratio vegetation index (RVI) and the green normalized vegetation index (GNDVI). Then, an analysis was performed for the correlation of these indices with rice and soybean yields, and yield estimation models were constructed using partial least squares regression.

Remote-sensing-based crop growth monitoring and yield estimation are mainly achieved by identifying characteristic parameters, analyzing the statistical relationships between crop growth and relevant metrics (e.g. LAI and biomass), and hence constructing estimation models. This crop growth assessment approach is simple, easy to use, and widely used. However, remote sensing information only reflects superficial physical conditions, and cannot truly reveal the internal processes and mechanisms of collective and individual crop growth, yield formation, and environmental interactions. Therefore, remote sensing methods lack explanatory mechanisms, and typically exhibit poor temporal and spatial expansion. In addition, because the acquisition of remote sensing data is often restricted by satellite revisit cycles and adverse weather conditions (clouds, rain and snow), continuous crop growth monitoring cannot be performed during the growing season, and hence the monitoring accuracy is limited to a certain extent. On the other hand, crop growth models are based on biophysical growth laws, meteorological conditions, and soil conditions. Also, these models use light, temperature, moisture and fertilization as driving factors, and take into account material balance and energy conservation matters. In fact, such models can be created based on computational and mathematical techniques to systematically and quantitatively express key processes in crop physiology including crop photosynthesis, respiration and transpiration. Indeed, dynamical mathematical models can be established to simulate the dynamic crop growth processes during the whole growth period with a fixed step. Therefore, computerized mathematical models with strong explanatory power have been widely used in crop growth simulation, physiological and biochemical index parameter inversion, yield estimation, etc^36–38^ used hyperspectral data within an analytical two-layer canopy reflectance model (ACRM) for LAI inversion in winter wheat. The results showed high LAI inversion accuracy with small model parameter uncertainty and appropriate band selection. Pan et al.^39^ exploited the PROSAIL model for LAI inversion in winter wheat, and showed high accuracy in the red-edge band using the cropping index. Also, Baret et al.^40^ combined artificial neural networks with the PROSAIL model for effective inversion of crop LAI.

Crop growth models are typically built on a regional scale for growth monitoring and yield estimation. However, such models overlook the large spatial and temporal variations in soil characteristics (such as soil moisture), crop parameters (such as LAI, biomass, and nitrogen content) and meteorological data^41^. Actually, uncertainties in these factors affect the physiological growth process and hence can lead to degradation in the accuracy of crop growth models. Those uncertainties can be reduced through the assimilation of remote sensing data which provides a great potential for accurate quantitative estimation of regional soil properties and canopy state variables. Also, the real-time acquisition and spatial continuity of remote sensing data can effectively enhance the applicability of crop growth models at regional scales. However, obtaining the input parameters of a crop growth model continuously quite is challenging. These challenges can be handled based on the respective advantages of remote sensing and crop growth models. Specifically, based on the data assimilation methodology, the remote sensing data is embedded into the relevant input parameters of the crop growth model or the correction model for local parameter adaptation, and thereby improving the accuracy of crop monitoring and yield estimation^37,42^. Yao et al.^43^ assimilated the MODIS LAI data into the BEPS crop growth model, and hence significantly improved the accuracy of corn yield estimation. Tripathy et al.^44^ assimilated LAI data retrieved by the SPOT satellite into the WOrld FOod STudies (WOFOST) crop growth model, and thus effectively improved the yield estimation accuracy for wheat and corn. Curnel et al.^45^ assimilated the LAI of winter wheat in the WOFOST model, optimized the model parameters using an ensemble Kalman filter, and realized regional-scale growth monitoring and yield estimation for winter wheat. Ma et al.^46^ assimilated MODIS-based LAI data into the WOFOST model, and improved the yield estimation accuracy by optimizing three model parameters, namely the date of emergence, initial biomass, and soil moisture content. Dente et al*.* and Silvestro et al.^47,48^ used remote sensing data obtained from ENVISAT, ASAR and MERIS for wheat LAI inversion, assimilated the data into the CERES-Wheat and AquaCrop models, and improved the model performance by adjusting model parameters such as the sowing time, wilting point, field water holding capacity, harvest index, temperature, and moisture.

The simple algorithm for yield estimation (SAFY) model is a crop growth model based on light-energy utilization theory. This model describes the biophysical crop processes (e.g. biomass accumulation, leaf distribution, leaf senescence, etc.) with empirical parameters, and hence simplifies the process of crop growth modeling. This model avoids the limitations of earlier models and is more applicable under universal conditions^49–51^.

This paper uses Sentinel-2 remote sensing data to retrieve the leaf area index (LAI) of winter wheat, assimilates the data into the SAFY crop growth model, and optimizes three sensitive model parameters using the SP-UCI optimization algorithm. Those parameters are the emergence date (D_0_), the effective light energy utilization rate (ELUE), and the senescence temperature threshold (STT). The paper basically seeks to create a dynamic model of winter wheat growth, estimate wheat LAI, biomass, dry aerial mass (DAM) and yield, and also use actual measurements to verify the estimation results. In essence, the paper provides new solutions for wheat crop growth monitoring and yield estimation using remote sensing, data assimilation, and crop growth modeling.

## Materials and methods

### Data acquisition and processing

#### Study area

**Figure 1:**
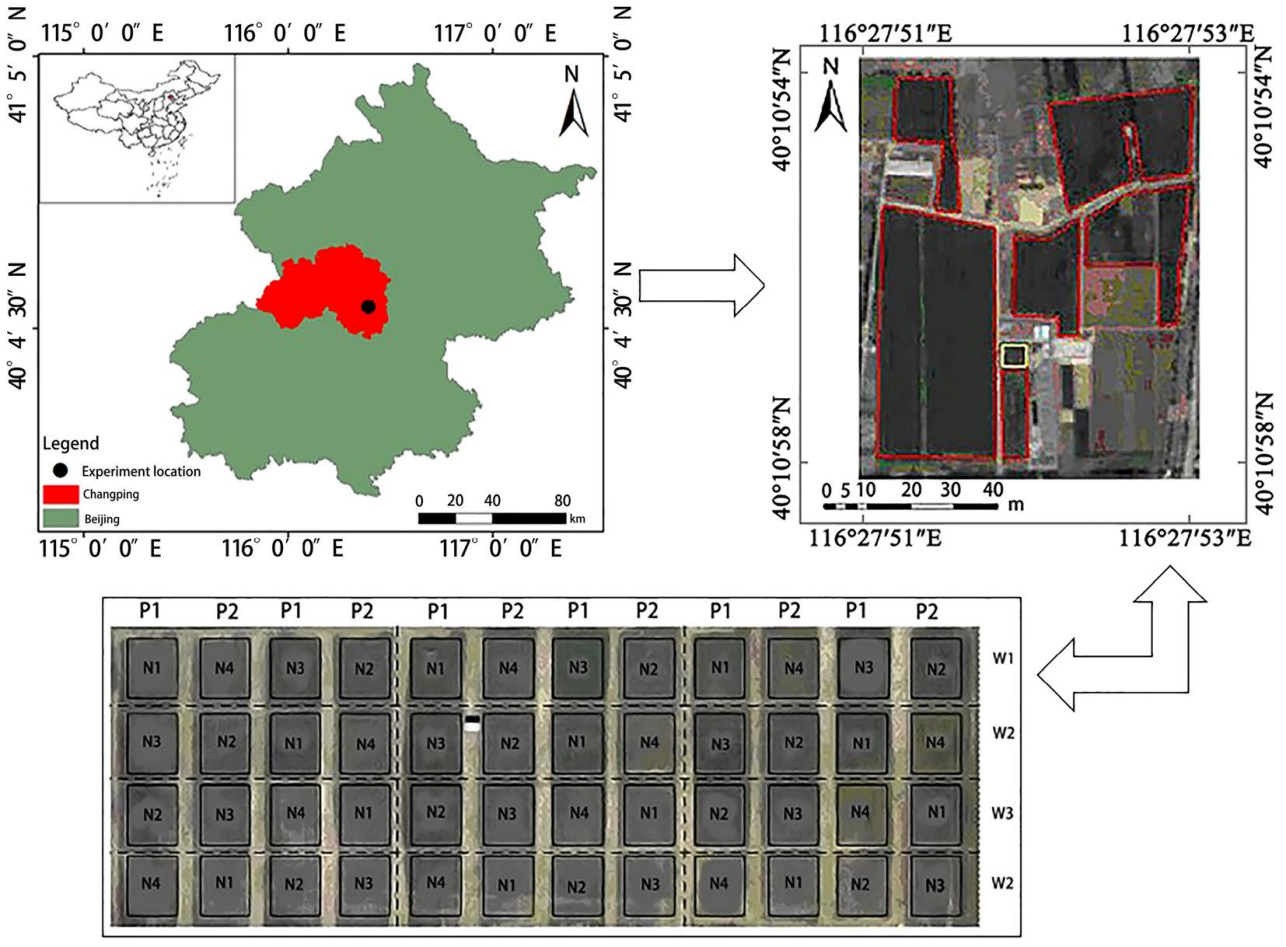
Study area and experimental design.

The study was conducted at the National Precision Agriculture Research Demonstration Base in Xiaotangshan Town, Changping District, Beijing. This area is located in the northeast of Xiaotangshan Town (116° 27′ 51″–116° 27′ 53″ E, 40° 10′ 48″–40° 10′ 54″ N) (Fig. 1), with an average elevation of 36 m above sea level. The area has a semi-humid continental climate in the northern temperate Monsoon Zone, and is characterized by high temperatures, rainy summers, cold and dry winters, and short springs and autumns, with an average frost-free period of 180 days throughout the year.

The study area has a total of 48 plots. The each plot size was 6 m^2^ with length 3 m and width 2 m. The sowing two wheat cultivars in 48 plots was done on November and harvesting was completed on June. The wheat planting density of each plot is 4.89 million plants/ha, and there are two wheat varieties, namely Jing 9843 (Variety 1, denoted by *P1*) and Zhongmai 175 (Variety 2, denoted by *P2*). The data was collected at turning green, jointing, flag picking, flowering, grain filling and harvest growth stages. As for the nitrogen fertilizer (urea) treatment, there are four possible settings: 0 kg/ha (*N1*), 195 kg/ha (*N2*), 390 kg/ha (N3), and 585 kg/ha (*N4*). Three levels of water treatment are possible: rain-fed (*W1*, No irrigation), normal water volume (*W2*, irrigation water volume of 146 mm), and doubled water volume (*W3*, irrigation water volume of 292 mm). Apart from the nitrogen fertilizer treatment, the base fertilizer is composed of 375 kg/ha of superphosphate and 150 kg/ha of potassium sulfate. The field management conditions of each community are the same.

#### Data acquisition and processing

The types of the collected data include satellite remote sensing data, the leaf area index, biomass, wheat yield, and meteorological data.

1. Remote sensing data acquisition

The remote sensing data was acquired by Sentinel-2 satellites, which is a high-resolution imaging satellite that carries a multispectral imager (MultiSpectral Instrument, MSI) with 13 bands, a width of 290 km, and a revisit period of 10 days. In this work, the remote sensing data was downloaded from the official website of the European Space Agency (ESA) (https://scihub.copernicus.eu/dhus/#/home). All image data are L1C products that have been ortho-rectified and geometrically corrected. We applied atmospheric data correction with the Sen2Cor software provided by ESA. A total of 4 data bands (B2, B3, B4, and B8) were obtained in the ENVI software (version 5.3).

In this paper, remote sensing images were obtained from the Sentinel-2 satellite through 7 phases with a relatively uniform time distribution. The acquisition times were: April 10, 2014, April 15, 2014, April 25, 2014, May 13, 2014, May 23, 2014, June 2, 2014, June 9, 2014, May 2, 2015, and May 9, 2015.

(2) LAI data acquisition

The LAI-2200C Plant Canopy Analyzer was used to collect LAI data according to user manual provide by CIMMYT^52^. For LAI measurement, we selected the plant side facing away from the sun, made one sky light measurement, and then placed the analyzer close to the plant root to measure 4 target values for each plot. The analyzer lens was kept upright, and finally the average value of the cell LAI was obtained. The upper side of fully sunlit leaves was chosen to perform three repeated measurements at each sample site. At each sampling site, the radiation value was first measured at the top of canopy, and then at four marking points under the canopy.

(3) Biomass data acquisition

For collecting biomass data in each plot we have followed CIMMYT user manual for field based traits measurement^52^. We selected three representative wheat plants, cut the above-ground plant parts, put the parts in a paper bag, and then put the bag in an oven. The temperature was set to 105 °C to kill bacteria. After 30 min, the temperature was set to 75 °C and the plant parts were dried to a constant weight for approximately 24–28 h. Then, the plant weight was measured with a balance. Finally, the biomass per unit area was calculated according to the plant density in the plot and the dry sample weight.

(4) Production data acquisition

When wheat became ripe, a certain area of wheat samples was harvested and recorded in each plot. The harvested wheat grains were then air-dried under natural conditions, and weighed with a balance. The yield per unit area was calculated according to the harvested area and the sample weight.

(5) Meteorological data acquisition

The meteorological data used in this work is the ERA-Interim data provided by the European Centre for Medium-Term Weather Forecast (www.ecmwf.int). The data includes the daily maximum temperature, the minimum temperature, and the daily radiation. The average of the daily maximum and minimum temperatures is the daily average temperature.

### Methods

1. Performance evaluation metrics
  The coefficient of determination (*R*^2^), the root-mean-square error (*RMSE*) and the normalized RMSE (*nRMSE*) were selected as performance evaluation metrics. These metrics are mathematically defined as:
  1 $$ R^{2} = \frac{{(\sum\nolimits_{i = 1}^{n} {y_{i} - \overline{y})^{2} } }}{{(\sum\nolimits_{i = 1}^{n} {x_{i} - \overline{y})^{2} } }}, $$
  2 $$ RMSE = \sqrt {\frac{{\sum\nolimits_{i = 1}^{n} {(x_{i} - y_{i} )^{2} } }}{n}} , $$
  3 $$ nRMSE = {{\sqrt {\frac{{\sum\nolimits_{i = 1}^{n} {(x_{i} - y_{i} )^{2} } }}{n}} } \mathord{\left/ {\vphantom {{\sqrt {\frac{{\sum\nolimits_{i = 1}^{n} {(x_{i} - y_{i} )^{2} } }}{n}} } {\overline{y}}}} \right. \kern-\nulldelimiterspace} {\overline{y}}}, $$
  where $x_{i}$ is the *i*th measurement, $y_{i}$ is the corresponding model estimate, $\overline{y}$ is the mean value of the model estimates, and *n* is the number of samples. Generally, the larger *R*^2^ is, the smaller the *RMSE* is, and the better the model fit is. The range of the *nRMSE* metric generally defines the model accuracy. A value of *nRMSE* < 10% indicates that the estimated and measured values are highly consistent, the range 10% < *nRMSE* < 20% indicates good consistency, while the range 20% ≤ *nRMSE* < 30% indicates medium consistency, and finally the range *nRMSE* ≥ 30% indicates poor consistency.
  (2) Construction of the vegetation index maps

Based on relevant research studies, six commonly used vegetation indices are selected, namely the enhanced vegetation index (EVI), the enhanced vegetation index 2 (EVI2), the modified simple ratio (MSR), the normalized vegetation index (NDVI), the optimized soil-adjusted vegetation index (OSAVI), and the ratio vegetation index (RVI). The mathematical definition of each of these indices is listed in Table 1.

**Table 1:** Mathematical expressions of various vegetation indexes.

Vegetation indices	Equation	References
EVI	${\text{EVI}} = 2.5\frac{{{\text{NIR}} - R}}{{{\text{NIR}} + 6R - 7.5B + 1}}$	^53^
EVI2	${\text{EVI2}} = 2.5\frac{{{\text{NIR}} - R}}{{{\text{NIR}} + 2.4R + 1}}$	^54^
MSR	${\text{MSR}} = \frac{{{\text{NIR}}/R - 1}}{{\sqrt {{\text{NIR}}/R} + 1}}$	^55^
NDVI	${\text{NDVI}} = \frac{{{\text{NIR}} - R}}{{{\text{NIR}} + R}}$	^56^
OSAVI	${\text{OSAVI}} = \frac{{{\text{NIR}} - R}}{{{\text{NIR}} + R + 0.16}}$	^57^
RVI	${\text{RVI}} = \frac{{{\text{NIR}}}}{R}$	^58^

R, G, B and NIR represent the reflectance of red, green, blue and near-infrared bands respectively.

(3) The SAFY crop growth model

The SAFY model is a crop growth model based on the theory of light energy utilization. This model simulates the daily dynamic changes of the crop LAI, DAM, and yield from the growth emergence to end. The daily radiation and daily mean temperature are necessary driving inputs for the SAFY model. In this model, crop growth is divided into two continuous stages, namely the growth stage and the senescence (or biological aging) stage. These stages are identified based on the accumulated summary temperature (SMT) after emergence.

From emergence to senescence, the crop biomass increases with photosynthesis. During this process, the aboveground biomass is calculated based on three factors, namely the temperature stress function, the absorbed photosynthetically active radiation (APAR), and the effective light use efficiency (ELUE). The product of these three factors gives the dry aerial mass (DAM) which is calculated as:

4 $$ DAM = ELUE \times F_{T} (Ta) \times APAR, $$

and the APAR factor is given by:

5 $$ APAR = (1 - e^{ - K * LAI} ) \times \varepsilon_{c} \times Rg, $$

where *R*_*g*_ is the daily radiation, $\varepsilon_{c}$ is the climate efficiency factor, *K* is the light interception coefficient, and $F_{T} (Ta)$ is the temperature stress function. This function is mathematically defined as

6 $$ F_{T} (Ta) = \left\{ \begin{gathered} 1 - \left( {\frac{{T_{opt} - T_{a} }}{{T_{opt} - T_{\min } }}} \right)\;^{2} \;\;\;\;\;\;T_{\min } < T_{a} < T_{opt} \; \hfill \\ 1 - (\frac{{T_{a} - T_{opt} }}{{T_{\max } - T_{opt} }})^{2} \;\;\;\;\;\;\;\;\;T_{opt} < T_{a} < T_{\max } \hfill \\ 0\;\;\;\;\;\;\;\;\;\;\;\;\;\;\;\;\;\;\;\;\;\;\;\;\;\;\;\;\;T_{a} < T_{\min } \;or\;T_{a} > T_{\max } , \hfill \\ \end{gathered} \right.\;\;\;\;\;\;\;\;\; $$

where *T*_*a*_ is the daily average temperature, *T*_*min*_, *T*_*opt*_, and *T*_*max*_ represent respectively the minimum, the most suitable, and the maximum temperatures for crop growth. If the ambient temperature is too high or too low, the growth rate of the crop biomass will decrease. When the daily average temperature is lower than *T*_*min*_ or higher than *T*_*max*_, the crop growth will stop.

In the growth stage, the crop leaves grow, and the biomass growth can be divided into two parts: leaf biomass growth and non-leaf biomass growth. The leaf biomass growth follows a distribution function (Eq. 8), and promotes a LAI increase (Eq. 9). When the accumulated temperature reaches the senescence temperature threshold (STT), the leaves enter the senescence stage at a specified rate (Eq. 10). When the LAI is less than 0.1, the senescence ends. The aforementioned quantities are defined as follows:

7 $$ SMT = \sum\nolimits_{{D_{0} }}^{t} {(T_{a} - T_{\min } )dt} , $$

8 $$ Pl = 1 - Pl_{a} \times e^{{Pl_{b} * SMT}} , $$

where *SMT* is the accumulated temperature, *D*_*0*_ is the crop emergence period, *Pl* is the proportion of biomass allocated to leaf tissues, while *Pl*_*a*_ and *Pl*_*b*_ are the distribution coefficients.

If *Pl* > 0, then

9 $$ \Delta LAI^{ + } = \Delta DAM \times Pl \times SLA, $$

where $\Delta LAI^{ + }$ is the daily *LAI* increment, and *SLA* is the specific area, i.e., the ratio of the leaf unit area to its dry weight.

If *SMT* > *STT*, then

10 $$ \Delta LAI^{ - } = LAI \times (SMT - STT)/R_{s} , $$

where $\Delta LAI^{ - }$ is the daily LAI decrease, *STT* is the senescence temperature threshold, and $R_{s}$ is the coefficient of leaf senescence.

The crop yield is expressed as

11 $$ GY = DAM_{\max } \times HI, $$

where $DAM_{\max }$ is the maximum aboveground biomass, and *HI* is the harvest index.

(4) SP-UCI parameter optimization algorithm adopted with SAFY

We used a modified version of the SAFY. This is a simple crop growth simulation model that has been successfully applied to evaluate crop growth and yield. This model can well summarize the biomass accumulation and allocation. Compared to popular crop growth models such as SAFY, DSSAT and WOFOST has few free parameters to specify, which makes it attractive for large-area practical applications. In SAFY, the aboveground dry biomass production is driven by incoming photosynthetically active radiation and constrained by air temperature. The environmental stress of crops was compensated for by the field-specific effective light use efficiency (ELUE) parameter. Water stress is an important factor limiting crop yield. Therefore, in this study, we used LAI and biomass to simulate the crop water stress dynamics. In this study, the data assimilation method adopted is the shuffled complex evolution with principal component analysis—University of California, Irvine (SP-UCI) is a global optimization algorithm for high-dimensional and complex problems^59^. Based on the SCE-UA algorithm, the SP-UCI algorithm combines a complex evolutionary algorithm, the simplex algorithm, and polynomial resampling to deal with particle-swarm search degradation in high-dimensional spaces^60^. Indeed, the SP-UCI algorithm enables efficient particle-swarm search in whole parameter spaces with high dimensionality.

Although many hyperparameters should be set in the SP-UCI algorithm, most of these hyperparameters are typically set following values recommended in earlier methods. The settings of the maximum number of runs (*maxn*) and the number of complexes (*m*) are problem-dependent. The SP-UCI optimization steps are as follows:

1. *Initialization* Under uncertain prior information, assume that the data samples follow a uniform distribution and that the optimization target is *n*-dimensional. Denote the number of evolved complexes by *m* (*m* ≥ 1) and the number of vertices selected within each complex by *p* (*p* ≥ *m* + 1). A total of *m* × *p* points are randomly sampled in the parameter range, and the function is evaluated at each point.
2. *Complex division *The sample points are randomly divided into *m* complexes, and each complex is sorted according to the value of the fitness function.
3. *Dimension monitoring and recovery *Principal component analysis is used to determine the particle swarm degradation level, and search for and recover the missing dimension.
4. *Complex evolution *Based on the modified competitive complex evolutionary (MCCE) algorithm, each complex is evolved by reflection, contraction, and mutation.
5. *Polynomial resampling *After the above steps, if a better evolution point is still not found, a point is randomly drawn following the multivariable normal distribution defined by the simplex.
6. *Convergence judgment *If the convergence criterion is met, the cycle is terminated. Otherwise, return to step (2).

(5) Partial least squares regression

Partial least squares regression (PLSR) is a regression method that combines features of set regression analysis, canonical correlation analysis, and principal component analysis. The PLSR method seeks to infer a linear relationship between the independent and dependent variables, solve the problem of multicollinearity, and also ensure model stability. The PLSR method can be described as follows. Suppose there are *p* independent variables $\left\{ {x_{1} ,x_{2} , \ldots ,x_{p} } \right\}$, Q dependent variables $\left\{ y \right\}$, and *n* samples altogether. Each sample has *p* independent variables and one dependent variable, with corresponding matrices X and Y. Firstly, the component *t*_*1*_ is extracted from X, which is a linear combination of $x_{1} ,x_{2} , \ldots ,x_{p}$. If a total of *m* components $t_{1} ,t_{2} , \ldots ,t_{m} (m < n)$ are extracted from X, and y is expressed through regression over the original independent variables, the regression of y to the components $t_{1} ,t_{2} , \ldots ,t_{m}$ is realized.

### Ethical approval

Permission was obtained for the collection of Wheat samples and the study complies with relevant national, international and institutional guidelines.

## Experimental results and analysis

### LAI inversion using Sentinel-2 remote sensing data

In the process of assimilating LAI remote sensing data into crop growth models, LAI inversion should be realized on the regional scale. At present, models for crop LAI inversion from remote sensing data are mainly divided into three categories: statistical models, mechanism models, and mixed models. Verrelst et al*.* and Cheng and Meng^61,62^ investigated the LAI inversion performance for statistical and mechanism models. The results showed that the statistical models can achieve higher LAI inversion accuracy than the mechanism ones. For statistical models, vegetation index methods are the most traditionally employed methods. Although such methods involve fewer crop-growth mechanisms, these methods are simple, easy to use, and can retrieve crop LAI in a timely and effective manner. Numerous studies have shown that optical remote sensing is effective for large-scale inversion of crop LAI, especially because the visible-light and near-infrared bands are highly correlated with crop LAI^63–65^. Therefore, we employed optical remote sensing data obtained from Sentinel-2 to retrieve wheat LAI using vegetation index methods. Based on the vegetation indices listed in Table 1, we randomly selected two thirds of the data samples (*n* = 256), used the PLSR method to construct the LAI inversion model, and finally calculated three performance metrics for each model (*R*^2^, *RMSE*, and *nRMSE*). The results are shown in Table 2.

**Table 2:** LAI inversion model and accuracy using Sentinel-2 remote sensing data.

Vegetation index	Model	Model accuracy		
		*R*^2^	*RMSE*	*nRMSE* (%)
EVI	$y = 8.48x - 1.64$	0.63**	0.84	25.80
EVI2	$y = 8.51x^{1.61}$	0.63**	0.83	25.50
MSR	$y = 1.26e^{0.36x}$	0.68**	0.78	24.00
NDVI	$y = 9.72x - 4.68$	0.62**	0.84	25.80
OSAVI	$y = 0.21e^{4.68x}$	0.69**	0.76	23.30
RVI	$y = 0.18x + 0.89$	0.69**	0.76	23.30

**Indicates the significance level of 0.01.

**Figure 2:**
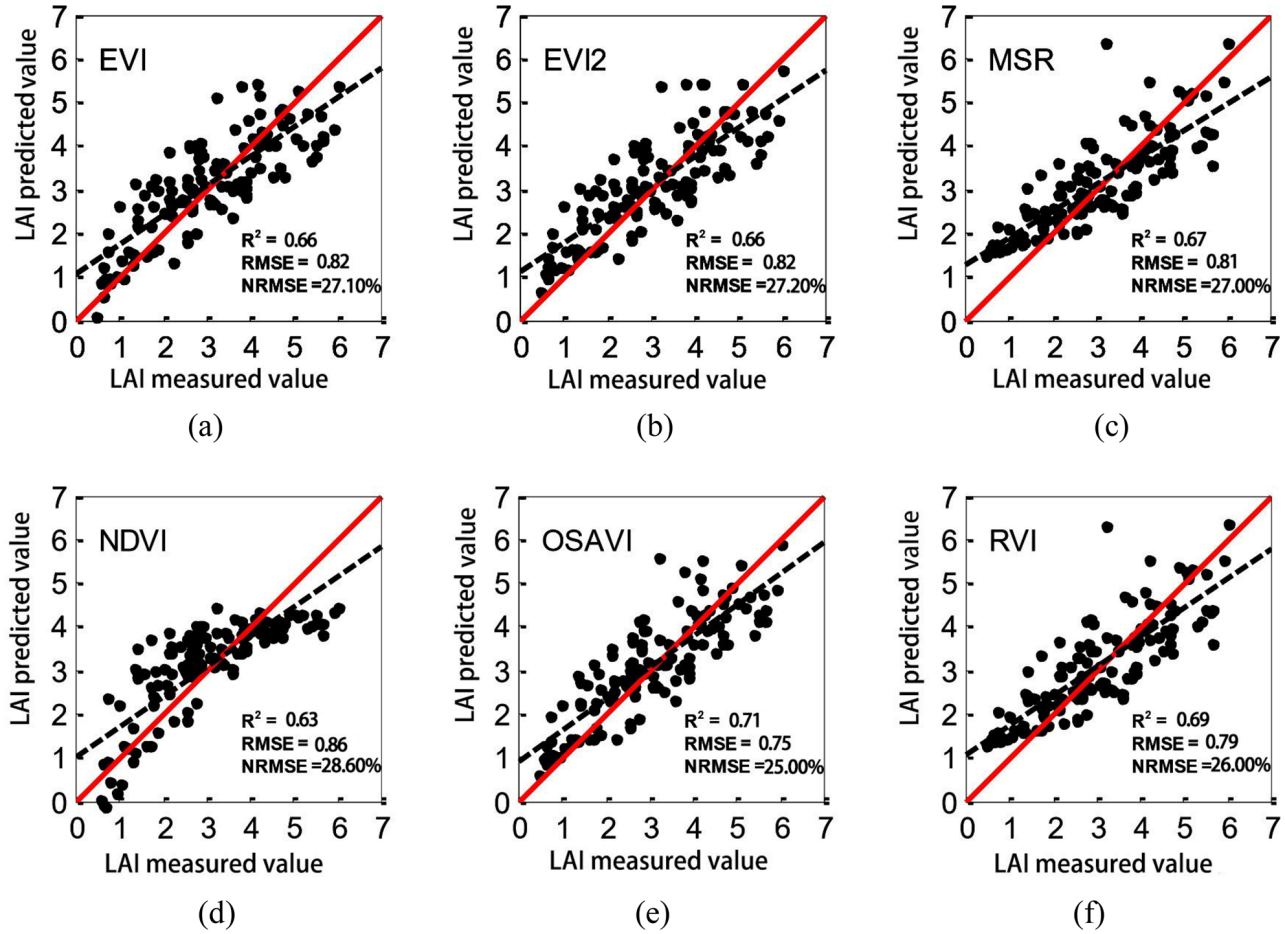
Scatter plots of predicted and measured LAI values for models with different vegetation indices.

In order to evaluate the stability and reliability of the LAI inversion model constructed by each vegetation index, the remaining one third of data samples (*n* = 128) (which were not used in the modeling process) were selected for checking the model accuracy. Pairs of LAI predicted and measured valued were used to create scatter plots for models with different vegetation indices, as shown in Fig. 2.

The results showed that the relationship between each vegetation index and the LAI was extremely significant (P < 0.01). According to the LAI estimation accuracy, the vegetation indices are ranked in a descending order as follows: OSAVI, RVI, MSR, EVI2, EVI and NDVI. Indeed, the OSAVI-based model had the highest accuracy, and the *R*^2^ values for the modeling and validation sets were 0.69 and 0.71, respectively. All other vegetation indices resulted as well in high modeling and validation accuracies, but the OSAVI model had the best performance and was hence chosen to retrieve LAI from Sentinel-2 remote sensing data.

### Estimation of wheat LAI, biomass, and yield with the SAFY model

#### Parameter settings for the SAFY model

Based on earlier studies^66–71^, the main parameters of the SAFY model were set as shown in Table 3.

**Table 3:** Main parameter settings for the SAFY model.

Parameter		Value	References
Parameter I	Climate efficiency factor $\varepsilon_{c}$	0.48	^66^
	Initial biomass DAM_0_	4.2 g/m^2^	^67^
	Optical interception coefficient K	0.5	^68^
	Three base point temperature (T_min_, T_opt_, T_max_)	0 °C/18 °C/26 °C	^69^
	Specific leaf area SLA	0.022 m^2^/g	^68^
	Recession coefficient R_S_	6875 °C/day	^68^
Parameter II	Partition function coefficient Pla	0.01–0.3	^70^
	Partition function coefficient Plb	10^–5^–10^–2^	^71^
Parameter III	Day after sowing D_0_		
	Effective light energy utilization ELUE	1.3–2.5 g/Mj	^71^
	Senescence temperature threshold STT	600–1500 °C	

The agricultural system complexity leads to uncertainties in crop growth parameters. When the parameters of a crop growth model are suitable for the area of interest, the model shows relatively high estimation accuracy. Otherwise, the model estimates will greatly deviate from the actual crop growth outcomes. Therefore, model parameter estimation and adjustment are crucial for ensuring the applicability of crop growth models in a certain area. In this paper, according to earlier methods^66,68,71^, the adjustable parameters of the SAFY model are divided into two categories: non-sensitive parameters (*Pla*, *Plb*) and sensitive parameters (D_0_, STT, ELUE). Based on the measured wheat LAI, DAM, and biomass data, a trial-and-error approach was followed to adjust the non-sensitive model parameters.

The wheat emergence date is generally about 10 days after sowing. During the flag picking period, the flag leaves grow. At that time, the wheat LAI reaches its maximum value, and the accumulated temperature is determined. The non-sensitive model parameters are adjusted as follows:

1. Use a trial-and-error method to determine the non-sensitive parameters (*Pla*, *Plb*). Firstly, the parameter *Pla* is set. According to Eq. (8), when the LAI reaches its peak value, the blade distribution function *Pl* is 0, and thus *Plb* is determined.
2. Initialize the parameters and simulate the SAFY model.
3. Construct the cost function based on the sum of squared errors between the measured and estimated LAI values:
  12 $$ J = \sqrt {\frac{{\sum\limits_{i = 1}^{n} {(S_{i} - M_{i} )^{2} } }}{n}} , $$
  where $M_{i}$ and $S_{i}$ respectively represent the measured and model estimated LAI values for the *i*th sample, and *n* represents the number of samples.
4. Terminate the iterations when either the objective function cannot be improved by 0.01% for 20 consecutive cycles, or the cost function is evaluated more than 10,000 times.
5. Estimate the error between the DAM and measured values.

The steps (1)–(5) are repeated until the DAM model estimate is close to the measured value. Finally, the non-sensitive parameters (*Pla*, *Plb*) are obtained as 0.16 and 0.0025, respectively. These parameters are fixed throughout the process of assimilating the remote sensing data into the SAFY model.

#### Wheat LAI, DAM and yield estimation

1. LAI estimation

**Figure 3:**
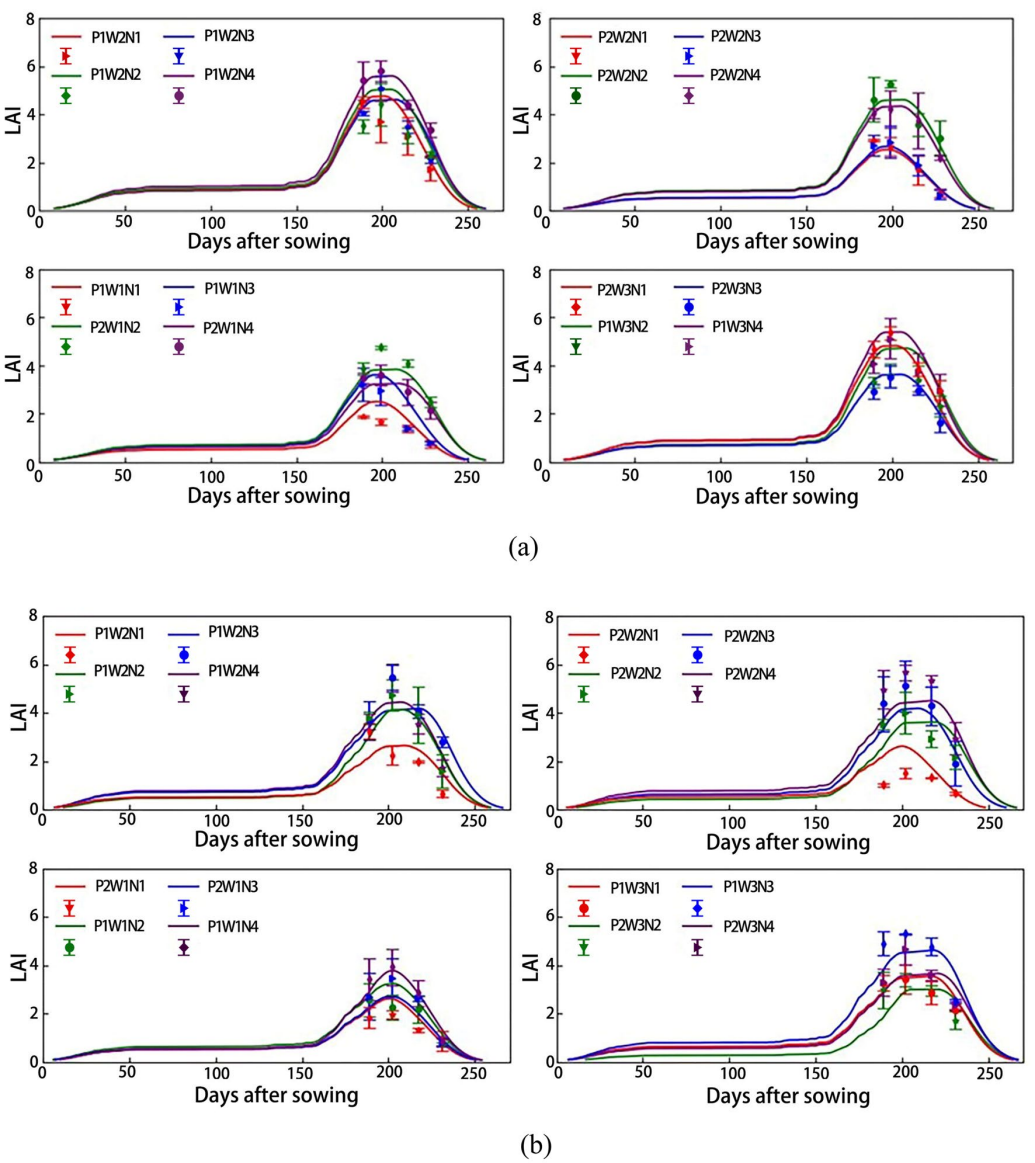
Relationships between estimated and measured values of wheat LAI for different wheat varieties and different irrigation and fertilizer settings in 2014 (**a**) and 2015 (**b**).

Using the Sentinel-2 remote sensing data, an OSAVI-based LAI inversion model was constructed. Then, optimization is carried out to get the best values of the three SAFY sensitive parameters, namely D_0_, ELUE, and STT for different test plots. The wheat LAI was thus estimated for different wheat varieties, and different irrigation and fertilizer settings. The relationships between the estimated and measured LAI values in 2014 and 2015 are inferred and shown in Fig. 3.

In Fig. 3, the error bars represent the actual LAI measurements in each growth period under different irrigation and fertilizer settings, while the curves represent the LAI estimated values. The results show that with the transition to later wheat growth periods, the SAFY model shows an increasing LAI trend. After the greening period (about 160 days after sowing), the LAI increases significantly, and at the end of wheat growth (about 200 days after sowing) the LAI reaches the maximum value and begins to decay approaching zero at the end of grouting (about 250 days after sowing). The estimated LAI values are in good agreement with the measured ones, indicating that the SAFY model can generally simulate the dynamic wheat growth and LAI progression during the entire growth period.

**Figure 4:**
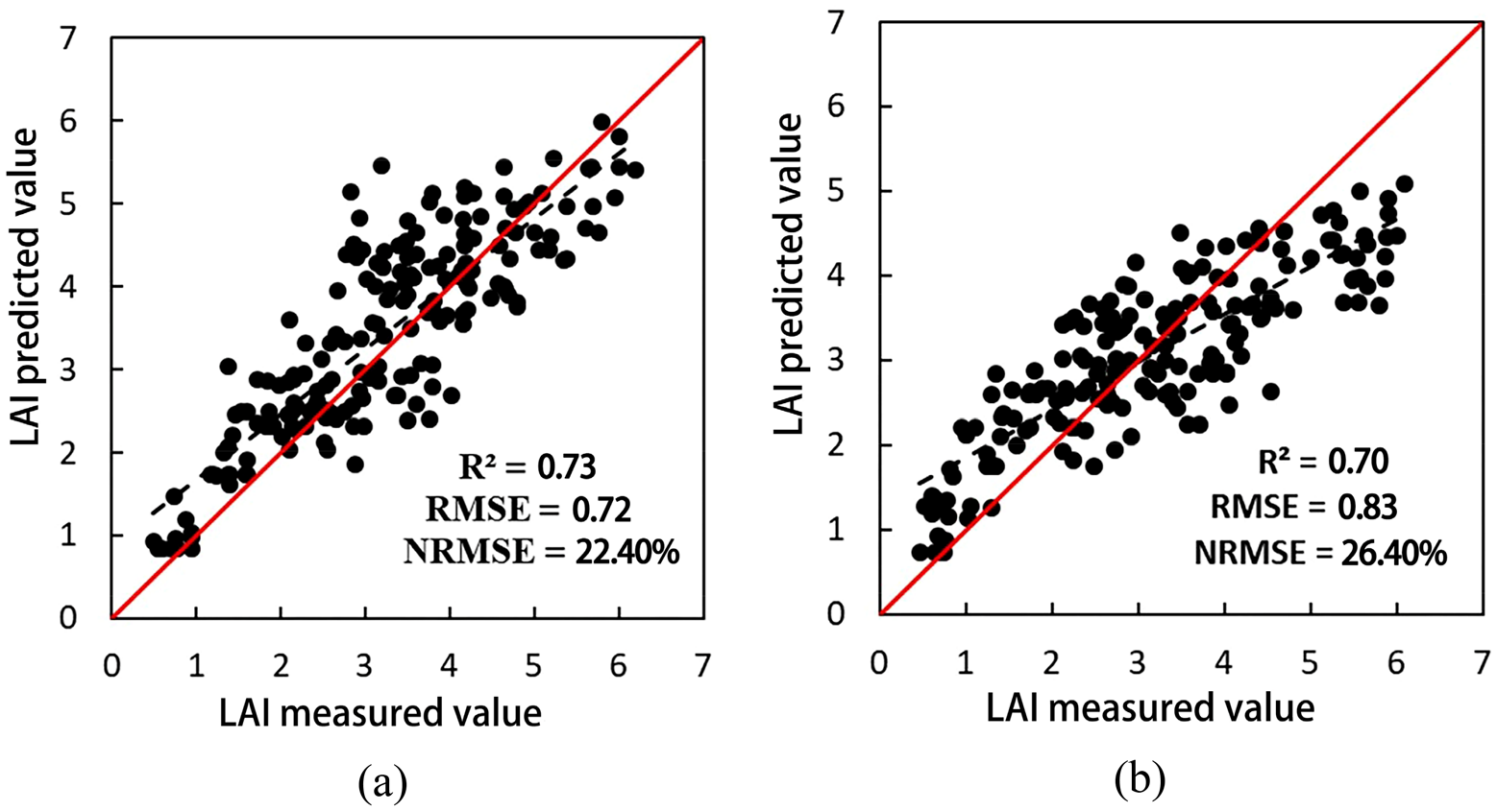
Scatter plots of the LAI estimated and measured values in 2014 (**a**) and 2015 (**b**).

We created scatter plots of the estimated and measured LAI values in 2014 and 2015 respectively, as shown in Fig. 4. In Fig. 4, the *R*^*2*^ values of the LAI estimation results for 2014 and 2015 are 0.73 and 0.70, respectively. These results show that the LAI estimated values are in good agreement with the measured ones. This indicates that the assimilation of remote sensing data into the SAFY model facilitates the effective estimation of the wheat LAI.

(2) DAM estimation

**Figure 5:**
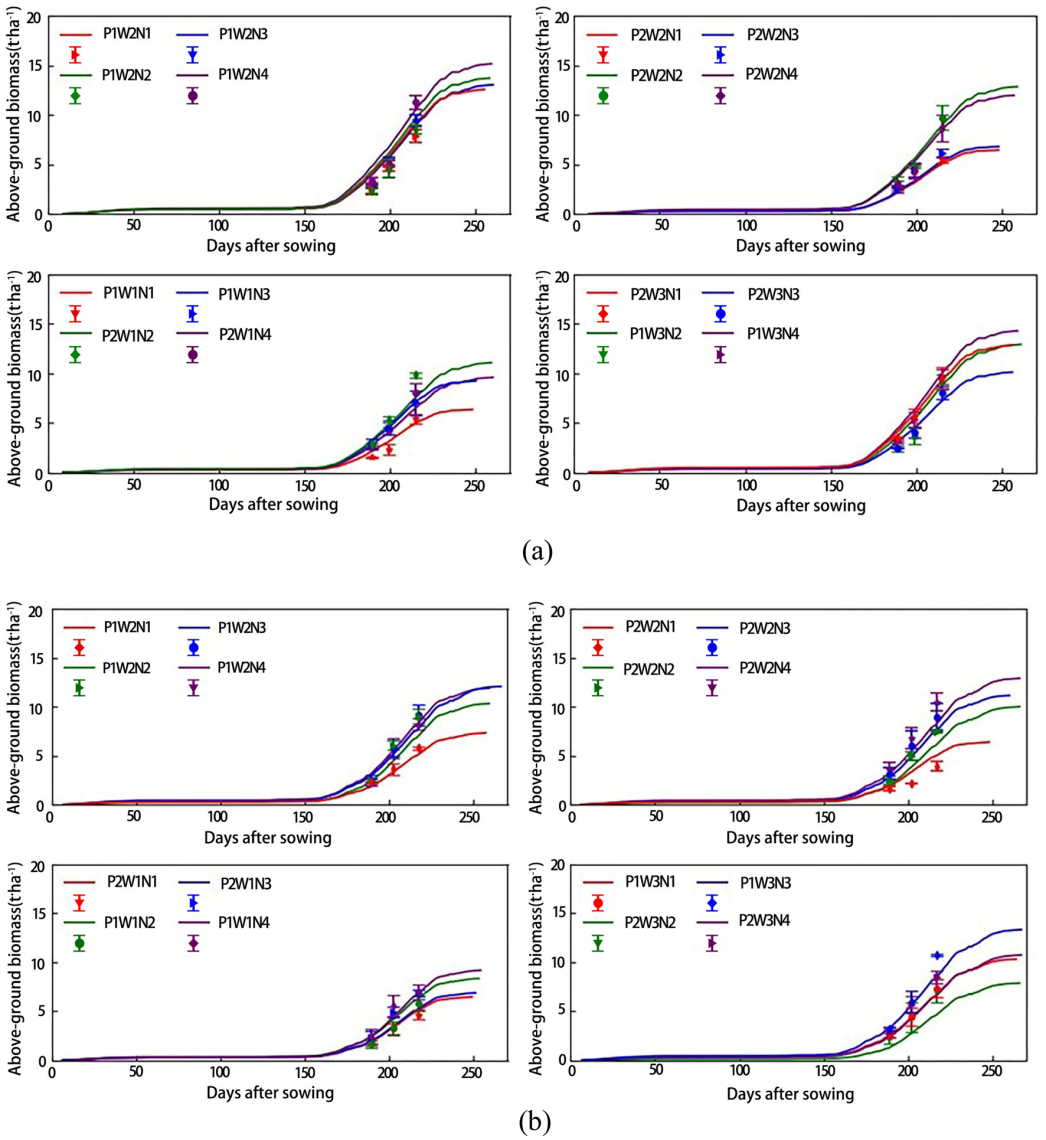
The relationships between the estimated and measured wheat DAM values for different wheat varieties and different irrigation and fertilizer settings in 2014 (**a**) and 2015 (**b**).

As mentioned above, using Sentinel-2 remote sensing data, the OSAVI-based LAI inversion model was constructed to optimize three sensitive parameters for different test plots: D_0_, ELUE, and STT. For each test plot, wheat DAM values were estimated for different wheat varieties and different irrigation and fertilizer settings. The relationships between the estimated and measured DAM values in 2014 and 2015 are shown in Fig. 5.

The results show that the SAFY-based wheat DAM estimate exhibits an overall increasing trend with time progression. After the greening period (about 160 days after sowing), the DAM growth rate increased significantly, and at the end of the growth period (about 200 days after sowing), the DAM growth rate reached its maximum, and then the growth gradually tended to be flat. At the end of the filling stage (about 250 days after sowing), the biomass growth ended. This shows that under different experimental conditions, the estimated DAM value is basically consistent with the measured one, and that the SAFY model can better model wheat DAM growth during the whole growth period.

**Figure 6:**
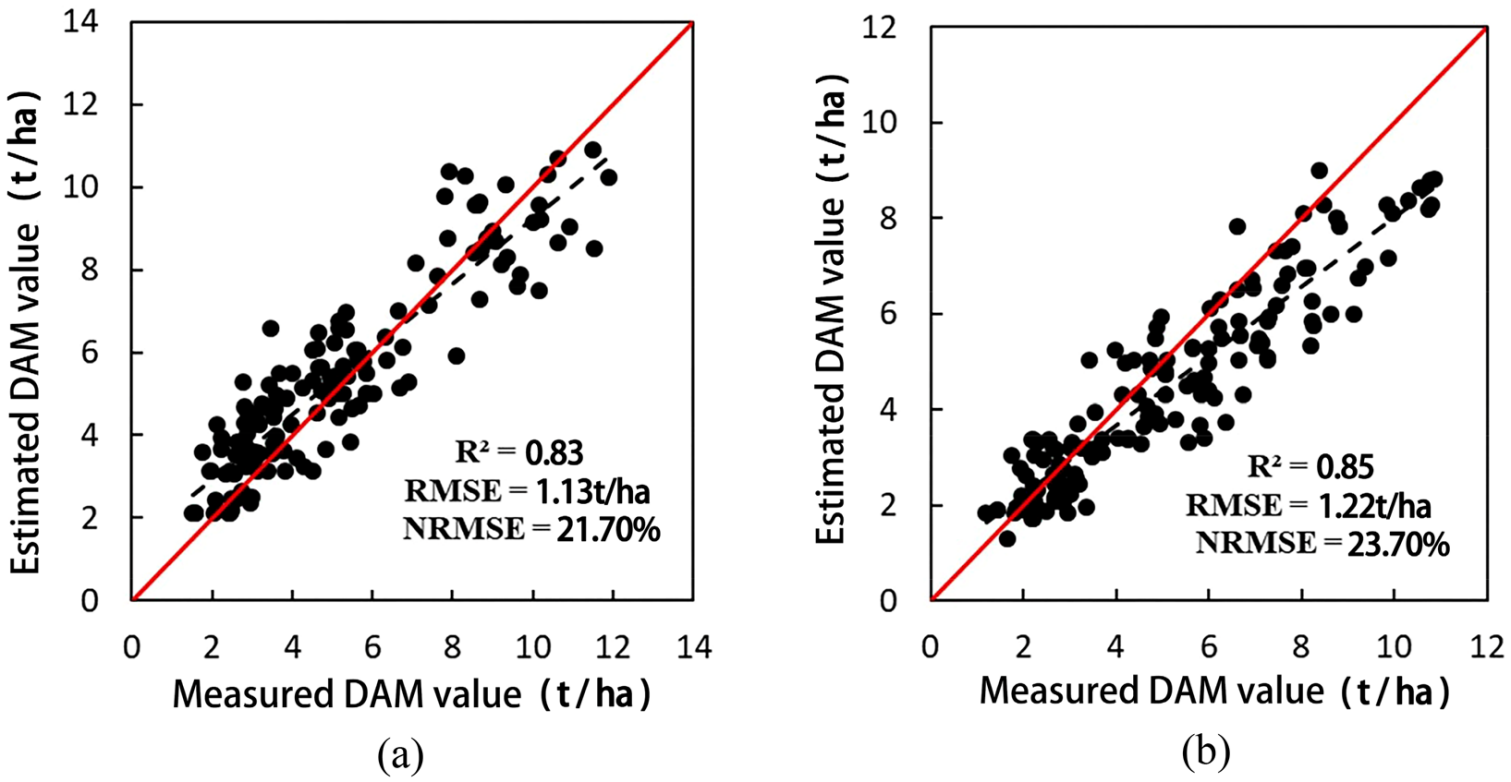
Scatter plots of the estimated and measured DAM values in 2014 (**a**) and 2015 (**b**).

Figure 6 shows scatter plots of the estimated and measured DAM values in 2014 and 2015 respectively.The estimated and measured DAM values in 2014 and 2015 resulted in *R*^2^ metrics of 0.83 and 0.85, respectively. Indeed, the estimated values are in good agreement with the measured ones, indicating that the assimilation of remote sensing data into the SAFY model helps with effective wheat DAM estimation.

(3) Production estimation

The harvest index (HI) is the ratio of the above-ground biomass (or DAM) to the yield when the crops are harvested. This index is an important parameter for crop yield estimation. Related studies show that the wheat HI is between 0.45 and 0.55 under non-stress conditions. Each of the irrigation, nitrogen fertilizers, and disease conditions can increase or decrease the HI value according to the condition timing and severity. Under adverse conditions, the HI can be reduced to 0.2–0.3 used the optimal HI for yield estimation^69^. The impact of water stress on biomass was directly considered and evaluated by simulating soil moisture^72–74^. The SAFY model can adequately take into consideration the effects of water stress in the LAI and ELUE simulations. Therefore, the HI value in this paper was set to 0.5.

**Figure 7:**
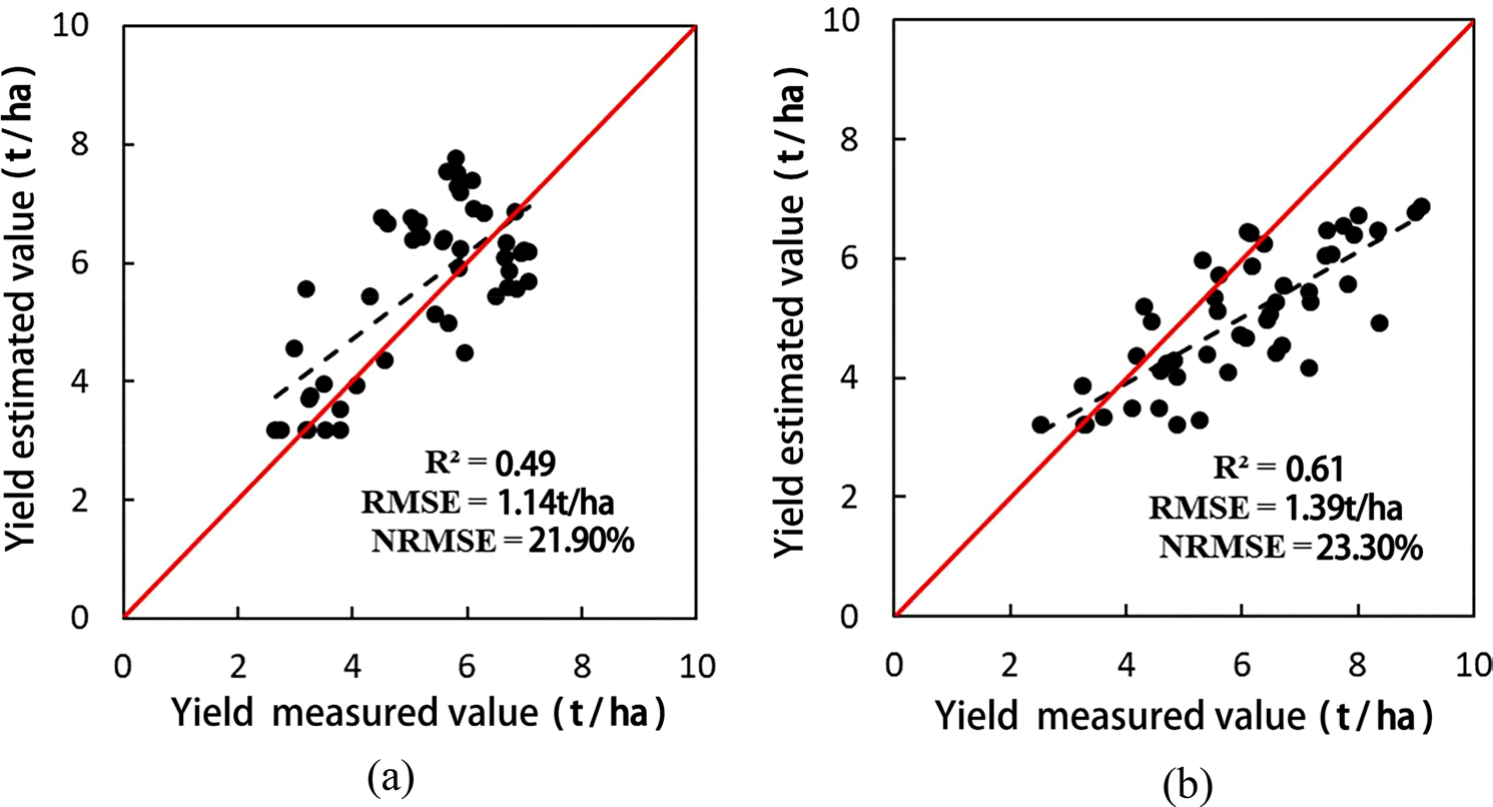
Scatter plots of the estimated and measured production values for 2014 (**a**) and 2015 (**b**).

Scatter plots of the estimated and measured wheat yields for 2014 and 2015 are shown in Fig. 7.The estimated *R*^2^, *RMSE*, and *nRMSE* metrics of wheat production in 2014 were 0.49, 1.13 t/ha, and 21.9%, respectively. For 2015, the corresponding metrics were 0.61, 1.38 t/ha, and 23.3%, respectively. However, the wheat production in 2014 was slightly overestimated, while that in 2015 was underestimated. In general, the estimated yield is in good agreement with the measured one, and this indicates that the SAFY model can reliably estimate wheat yield.

## Discussion

The remote sensing technologies have desirables with characteristics of being objective, rapid, non-destructive, and of wide coverage area^63^. Numerous in-depth studies have been conducted on the use of remote sensing data for crop growth monitoring, parameter retrieval, and yield estimation. However, remote sensing data only obtains instantaneous and superficial physical crop conditions, while this type of data cannot effectively reveal the mechanisms of environmental, soil, and farmland processes (including irrigation, and fertilization) and their effects on crop growth and yield formation^63–65^. So, models based only on remote sensing data suffer from poor universality. In addition, existing studies have shown that as crops grow, the remote sensing coverage gradually increases, spectral saturation effects become more visible, and parameter inversion becomes far from ideal^75^.

Alternatively, crop growth models have the advantages of possessing strong mechanisms and anti-interference capabilities. Therefore, many methods have been proposed to use crop growth models for parameter inversion and yield estimation. However, if such models were developed for limited areas, then the models may not scale well as the spatial scale increases^50^. Consequently, model parameters would be difficult to estimate with spatial variations, the model accuracy would deteriorate, and the models wouldn’t show good generalization performance. Therefore, we assimilated remote sensing data into crop growth models i.e. SAFY to achieve complementary advantages of both approaches, and hence improve the model universality. On the one hand, such assimilation can provide crop information with ‘ground-truth values’ to assist in correcting model deviations. The assimilation also effectively reduces the difficulty of regional model construction in terms of the initial conditions and model parameters. On the other hand, the resulting enhanced models allow comprehensive investigation of the inherent mechanisms of crop growth and development^51^.

Compared with DASST and WOFOST, SAFY is relatively simple. Therefore, SAFY has a poor capability of correcting LAI. The accuracy of yield estimation by SAFY and LAI through data assimilation depends on the inversion accuracy of LAI. Moreover, in the late growth stage of crops, there will be a phenomenon of vegetation supersaturation^50,51,71^. This will lead to a decrease in the accuracy of LAI inversion for crops. Especially in the linear model, the actual LAI of crops would be higher than the inversion LAI. This can affect the accuracy of production estimation. Moreover, In comparison with other crop growth models^72–74^, the SAFY crop growth model exploits the theory of light energy efficiency, and overcomes limitations arising from the numerous and complicated parameters of other models. Defects that are difficult to determine simplify the process of crop growth modeling, maintain the advantages of crop growth models, and are more applicable under universal conditions. In this paper, remote sensing data and the SP-UCI optimization method are used to optimize the key parameters in the SAFY model. The average coefficients of determination *R*^2^ of the LAI, biomass and yield estimates are 0.72, 0.84 and 0.55, respectively. The corresponding average *RMSE* values are 0.77, 1.17 g, and 1.26 kg, respectively. Also, the average *nRMSE* values are 24.4%, 22.7% and 22.6%, respectively. The model estimates are in good agreement with the measurements and are in line with the actual wheat growth. This indicates that the proposed crop growth model with assimilated remote sensing data can be used for estimating the LAI and biomass of winter wheat. The model estimates are feasible, and consistent with those of earlier studies^71^. The low *R*^2^ for yield estimation was due to high error probability in harvest index (HI) parameter, that might be because of small sample size for the estimation HI.

In comparison to actual measurements in 2014 and 2015, our model seems to overestimate the LAI and biomass values, especially for 2014. This tendency may be due to the lack of remote sensing wheat data for the pre-winter period, and also the previous assimilation windows that are relatively stable and directly caused by assimilation modeling.

The performance of the SP-UCI optimization method with the SAFY model, was comparable with the results of others upgrading assimilation method encountered in literature^72^, which is especially encouraging when considering that, despite the limited number of plots and sampling size error of LAI estimation of 22 to 26%, the error on the yield estimation was around 21 to 23%. This allows the application of this upgraded assimilation method in future wheat breeding programs. The encouraging results of the present work obtained from SP-UCI optimization method with SAFY should be confirmed with further studies with other validations, possibly with experiments that provide more frequent field measurements, a larger variety of climatic and environmental datasets and a higher quality of remote sensing data collection.

## Conclusions

In this paper, the SP-UCI optimization algorithm is used to assimilate remote sensing spectral data into the SAFY crop growth model, build an assimilation system, and estimate three key wheat growth parameters (the leaf area index, biomass, and yield). The reliability, accuracy, and robustness of the assimilation system were evaluated and verified based on the winter wheat growth data under different irrigation and fertilizer settings for two consecutive years. The estimated LAI, biomass and yield were consistent with the actual measurements. This indicates the feasibility of the proposed assimilation scheme in crop growth monitoring and yield estimation.

There is an immediate need to continue this research in several directions. First, we note that this study is based on known field data in the study area. For model simulation, the starting point of the simulation, i.e., the day of emergence, is a known quantity. However, when the assimilation model is applied on the regional scale, the emergence date of each crop is generally unknown. To achieve truthful and complete crop growth monitoring, the date of emergence must be first determined. Wheat growth is highly sensitive to the sowing date. A date that is too early or too late will seriously affect the late crop growth and the early emergence rate. Therefore, specifying the emergence date on the regional scale is crucial for accurate growth modeling. At the same time, the proposed model didn’t account for the effect of the irrigation conditions on the harvest index when estimating the final yield. Indeed, the model parameters need further optimization to ensure model robustness under variations in field conditions.

Another area that warrants future work pertains to the process of the assimilation of remote sensing data into crop growth models for yield estimation. The remote sensing data is mainly used to optimize the sensitive model parameters, which have a great influence on crop growth. That is, remote sensing data reflects the influence of several factors that affect crop yield. These factors include weather conditions, wheat variants, and soil characteristics. Crop yield is also affected by field management practices which are essentially based on the joint effects of the aforementioned factors. In addition to the sensitive model parameters, the fixed values of other insensitive model parameters will also affect the crop yield estimates. However, some parameters may not have good regional representation due to limited data availability. In the future, data types could be further increased to improve the representativeness of model parameters.
